# High-Serum Angiopoietin-Like Protein 3 Levels Associated with Cardiovascular Outcome in Patients with Coronary Artery Disease

**DOI:** 10.1155/2020/2980954

**Published:** 2020-03-27

**Authors:** Ming-Chun Chen, Bang-Gee Hsu, Chung-Jen Lee, Ji-Hung Wang

**Affiliations:** ^1^Department of Pediatrics, Hualien Tzu Chi Hospital, Buddhist Tzu Chi Medical Foundation, Hualien, Taiwan; ^2^School of Medicine, Tzu Chi University, Hualien, Taiwan; ^3^Division of Nephrology, Hualien Tzu Chi Hospital, Buddhist Tzu Chi Medical Foundation, Hualien, Taiwan; ^4^Department of Nursing, Tzu Chi University of Science and Technology, Hualien, Taiwan; ^5^Division of Cardiology, Hualien Tzu Chi Hospital, Buddhist Tzu Chi Medical Foundation, Hualien, Taiwan; ^6^Cardiovascular Research Centre, Hualien Tzu Chi Hospital, Buddhist Tzu Chi Medical Foundation, Hualien, Taiwan

## Abstract

**Background:**

Angiopoietin-like protein 3 (ANGPTL3) plays a pivotal role in lipid metabolism and angiogenesis, and there is growing interest regarding the association between ANGPTL3 and coronary artery disease (CAD). This study aims to investigate whether ANGPTL3 levels can be used to predict the future occurrence of major adverse cardiovascular events (MACEs) in patients with CAD.

**Methods:**

Overall, 90 patients with CAD were enrolled between January and December 2012. The study's primary endpoint was incidence of MACEs. Patient follow-up was completed on June 30, 2017.

**Results:**

Following a median follow-up period of 54 months, 33 MACEs had occurred. Patients reporting MACEs had lower statin use (*P*=0.022) and higher serum C-reactive protein (*P* < 0.001) and serum ANGPTL3 (*P* < 0.001) levels than those without MACEs. Kaplan–Meier analysis revealed higher cumulative incidence of CV events in the high ANGPTL3 group (median ANGPTL3 level ≥ 222.37 ng/mL) than in the low ANGPTL3 group (log-rank *P*=0.046). Multivariable Cox regression analysis demonstrated that ANGPTL3 levels were independently associated with MACEs in patients with CAD (hazard ratio: 1.003; 95% confidence interval: 1.000–1.005; *P*=0.026) after adjusted for age, gender, and body mass index, classical risk factors, and potential confounders.

**Conclusions:**

Serum ANGPTL3 levels could serve as a biomarker for future occurrence of MACEs in patients with CAD.

## 1. Introduction

Coronary artery disease (CAD), a significant health problem and global burden, is a leading cause of disability and death worldwide [1]. In developing countries, CAD-related deaths were estimated to be as high as 17.5 million in 2005 and are further expected to increase by 137% in males and 120% in females by 2020 [2]. Patients with CAD are typically asymptomatic initially, and major adverse cardiovascular events (MACEs) are more likely to occur in those presenting with severe CAD and significant clinical conditions, including myocardial infarction (MI), cardiac arrest, stroke, or death from cardiovascular (CV) events [3]. Therefore, it is important to identify biomarkers that may be early indicators of CAD or MACEs and further strengthening preventive strategies.

Angiopoietin-like protein 3 (ANGPTL3) is a 70 kDa 460-amino acid long secretory glycoprotein primarily expressed in the human liver [4]. It can be detected in systemic circulation and has been implicated in angiogenesis and atherogenesis; currently, it is regarded as an endocrine signaling factor [5–8]. ANGPTL3 can regulate serum lipid levels by acting on lipoprotein lipase- (LPL-) and endothelial lipase- (EL-) mediated triglyceride (TG) and phospholipid hydrolysis [9]. An inherited disorder of familial combined hypolipidemia with complete ANGPTL3 deficiency was associated with protection from CAD due to absence of coronary atherosclerotic plaque [10, 11]. ANGPTL3 plays important roles in lipid and lipoprotein trafficking and metabolism, affecting lipid and glucose metabolism homeostasis [12, 13]. Moreover, ANGPTL3 has demonstrated a positive correlation with CV risk assessment parameters of carotid and femoral artery intima-media thickness in healthy human subjects after adjusting for classical risk factors [14]. In a previous study, we have shown a positive association between serum ANGPTL3 levels and aortic augmentation index values in patients with CAD [15]. Dewey et al. have reported that patients with heterozygous, loss-of-function (LOF) ANGPTL3 variants had significantly lower serum TG, low-density lipoprotein cholesterol (LDL-C), and high-density lipoprotein cholesterol (HDL-C) levels than those without these variants [16]. Furthermore, in dyslipidemic mice treated with an ANGPTL3-inhibiting human monoclonal antibody further decreased in the atherosclerotic lesion area than the control group [17].

Although evidence is accumulating of an association between ANGPTL3 and CAD, the association between serum ANGPTL3 levels and long-term CV outcomes in patients with CAD has rarely been reported [11, 16, 18]. Therefore, we conducted this study to determine the association between serum ANGPTL3 levels and MACEs in patients with CAD.

## 2. Methods

### 2.1. Patients

Overall, 90 participants with CAD visiting the CV outpatient department of Buddhist Tzu Chi General Hospital, Hualien, Taiwan, were recruited between January and December 2012. This study was approved by the Protection of Human Subjects Institutional Review Board of Tzu Chi University and Hospital. After reviewing patients' medical records, those with >50% stenosis in any segment following coronary angiography were identified as having CAD. Using standard mercury sphygmomanometers with appropriate cuff sizes, morning blood pressure levels were measured by trained staff on the right arm of all study participants after a minimum 10 min rest. Systolic and diastolic blood pressure were measured thrice at 5 min intervals and averaged for analysis. We defined hypertension according to the Eighth Joint National Committee (JNC 8) guidelines (SBP ≥ 140 mmHg and/or DBP ≥ 90 mmHg or receiving any antihypertensive drugs in the past 2 weeks). Patients were diagnosed with diabetes mellitus (DM) if fasting plasma glucose levels were ≥126 mg/dL or were undergoing oral hypoglycemic medications or insulin therapy [19]. All participants were asked to provide a signed informed consent form before the investigation. Only patients from the CV outpatient department with a CAD history were included. Participants with acute infections, acute MI, or pulmonary edema during blood sampling and those who refused to provide informed consent were excluded.

### 2.2. Anthropometric Analysis

Patient weight and height were measured in light clothing without shoes (adjusted to nearest 0.5 kg and 0.5 cm, respectively), and body mass index (BMI) was calculated using Quetelet's formula (weight (kg)/height (m^2^)) [16, 20, 21].

### 2.3. Biochemical Investigations

After an 8 h overnight fasting, approximately 5 mL blood was sampled from all participants and immediately centrifuged at 3000 g for 10 min. Serum blood urea nitrogen (BUN), creatinine, fasting glucose, TG, total cholesterol (TCH), HDL-C, LDL-C, and C-reactive protein (CRP) levels were determined using an autoanalyzer (COBAS Integra 800, Roche Diagnostics, Basel, Switzerland) [16, 20, 21]. Serum ANGPTL3 (R&D Systems, Inc., Minneapolis, MN) levels were quantified using the commercial enzyme-linked immunosorbent assay [16]. The intra-assay and interassay coefficients of variation in the measurement for ANGPTL3 were 4.1% and 6.7%, respectively. Estimated glomerular filtration rate (eGFR) was calculated using the Chronic Kidney Disease Epidemiology Collaboration equation.

### 2.4. CV Event Monitoring

This study's primary endpoint was the incidence of MACEs, including cardiac death, cardiac arrest, MI, stroke, nonfatal stroke or other arterial thrombotic events, and hospitalization from CV conditions, such as unstable or progressive angina and heart failure. Follow-up time (months) was estimated after the last hospital outpatient or inpatient record was reviewed or the last telephone interview was conducted (June 30, 2017). Moreover, event time (months) was estimated when the first MACE occurred. Patient follow-up was conducted by a study nurse who was blinded for participants' baseline measurements and study protocol.

### 2.5. Statistical Analysis

Data were coded and analyzed using the Statistical Package for Social Sciences (SPSS) version 19.0 (SPSS Inc., Chicago, IL, USA) software. Variable distribution pattern was analyzed with the Kolmogorov–Smirnov test. Normally distributed variables were expressed as mean ± standard deviation, and patient comparisons were performed using Student's independent *t*-test (two-tailed). Data not normally distributed were expressed as median and interquartile range, with patient differences compared using the Mann–Whitney *U* test (TG, fasting glucose, BUN, creatinine, CRP, and ANGPTL3). Data expressed as the number of patients were analyzed using the chi-squared test. Kaplan–Meier survival curves with a log-rank test were used to estimate event-free survival during follow-up based on median ANGPTL3 levels. Cox regression models were used to examine factors associated with CV events. A *P* value of <0.05 was considered significant.

## 3. Results

Demographic, clinical, and biochemical characteristics of the 90 patients with CAD are shown in Table 1. Overall, 44 (48.9%) and 70 (77.8%) patients had DM and hypertension, respectively. The high ANGPTL3 group (median ANGPTL3 level > 222.37 ng/mL) showed significantly higher serum CRP levels than the low ANGPTL3 group (median ANGPTL3 level ≤ 222.37 ng/mL; *P* < 0.001). Patients reported the use of the angiotensin-converting enzyme inhibitor (ACEi; *n* = 28; 31.1%), angiotensin-receptor blockers (ARB; *n* = 36; 40.0%), *β*-blockers (*n* = 54; 60.0%), calcium-channel blockers (CCB; *n* = 30; 33.3%), statins (*n* = 67; 74.4%), and fibrate (*n* = 16; 17.8%). No significant differences were found between ANGPTL3 groups considering age, sex, BMI, BP, DM, or hypertension comorbidities or ACEi, ARB, *β*-blockers, CCB, statins, or fibrate use.

After a median follow-up of 54 months, 33 CV events were reported. Patients with CV events had higher ANGPTL3 (*P* < 0.001), CRP (*P* < 0.001) levels, and severity of baseline CAD (*P* < 0.001) and lower statin use (*P*=0.022) than those without CV events. No significant differences in age, sex, DM, or hypertension comorbidities or ACEi, ARB, *β*-blockers, CCB, or fibrate use were observed between patients with and without CV events (Table 2).

**Figure 1:**
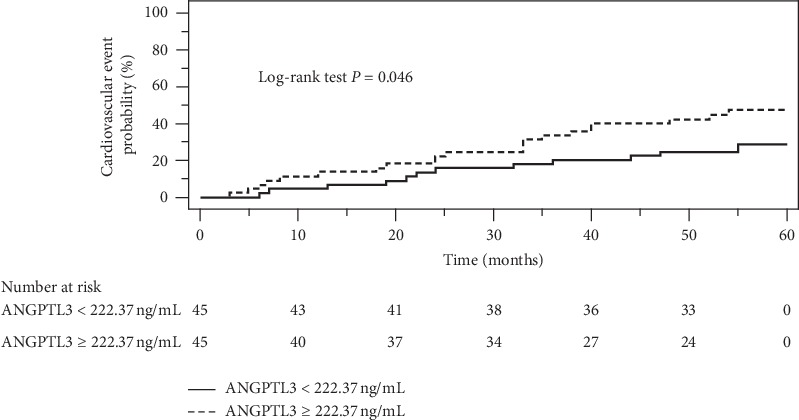
Kaplan–Meier analysis of cardiovascular events in 90 patients with coronary artery disease according to median serum angiopoietin-like protein 3 (ANGPTL3) levels.

Kaplan–Meier analysis revealed higher cumulative incidence of CV events in the high than in the low ANGPTL3 group (log-rank *P*=0.046; Figure 1). In patients with CAD, the unadjusted and Cox regression analysis of ANGPTL3 levels with other factors associated with CV events is presented in Table 3. In CAD patients, ANGPTL3 remained a significant predictor of the increased risk for CV events (unadjusted hazard ratio (HR) per increase of ANGPTL3 by 1 ng/mL: 1.003, 95% confidence interval (CI): 1.002–1.004; *P* < 0.001). ANGPTL3 remained significantly associated with an increased risk for CV events following adjustment for age, gender, and BMI (adjusted HR 1.003, 95% CI: 1.002–1.005; *P* < 0.001) as well as following additional adjustment for DM, hypertension, fasting glucose, TCH, TG, LDL-C, eGFR, statin used, serum CRP level, and severity of baseline CAD (adjusted HR 1.003, 95% CI: 1.000–1.005; *P*=0.026).

## 4. Discussion

This study reveals higher fasting ANGPTL3 levels that developed MACEs in patients with CAD during follow-up, and serum ANGPTL3 levels are independently associated with an increased risk of MACEs in these patients.

Previous studies have reported that inflammation and dyslipidemia are pivotal contributors to initiation and progression of coronary atherosclerosis [22–24]. Systemic inflammatory status is positively associated with severity of CAD, and CRP is a well-established biomarker of inflammation [22, 25]. The present study confirms that patients with CAD having high-serum ANGPTL3 levels have significantly higher CRP values than those with low ANGPTL3 levels. Patients with CAD who developed new MACEs had significantly higher CRP levels than those without MACEs during the follow-up period. Although no direct evidence of ANGPTL3-induced inflammation exists, certain studies have indicated that other ANGPTL family members, such as ANGPTL2, promote chronic adipose tissue inflammation and plasma CRP positively correlating with plasma ANGPTL4 in patients with metabolic syndrome and type 2 diabetes [26, 27]. Further studies are necessary to investigate the precise mechanisms of ANGPTL3 and inflammation in humans.

ANGPTLs are important modulators of lipoprotein metabolism and potential targets for CV disease risk regulation [17]. Animal studies have shown that deletion of *Angptl3* can reduce atherosclerosis development in apolipoprotein *E* knockout mice [7]. Higher circulating ANGPTL3 levels were observed in patients with CAD compared with healthy controls [11]. In the study by Stitzielet al., three individuals with complete ANGPTL3 deficiency due to heterozygous ANGPTL3 LOF mutations demonstrated no evidence of coronary atherosclerotic plaque compared with matched first-degree relative controls without ANGPTL3 LOF mutations [11]. Whole-exome sequencing analysis of 58,335 participants from the DiscovEHR study and 130,483 participants from four human genetic cohorts (including Duke Catheterization Genetics cohort, Copenhagen General Population Studies, the University of Pennsylvania Medicine BioBank, and the Taiwan Metabochip consortium) revealed that heterozygous ANGPTL3 LOF carriers with approximately 50% lower serum ANGPTL3 levels than noncarriers had a 39% lower probability of CAD [17]. A recent study has shown that patients with the lowest circulating ANGPTL3 levels (mimicking pharmacological inhibition of ANGPTL3) had a 35% reduced risk of MI compared with those with highest levels [11]. In our previous study, circulating ANGPTL3 levels positively correlated with aortic augmentation index values (a marker of arterial stiffness significantly associated with CAD degree) among patients with CAD, even after adjusting for confounding factors [16]. The present study corroborates that patients with CAD who developed MACEs have significantly higher ANGPTL3 levels than patients without MACEs. These findings indicate that elevated serum ANGPTL3 level is an independent risk factor for CV events in populations with established CAD and suggest that including ANGPTL3 in a CV risk model may increase the predictive power for early detection of MACEs.

In multivariable Cox regression analysis, elevated ANGPTL3 levels independently increased the risk of MACEs in patients with CAD. The mechanism underlying the induction of adverse CV event by ANGPTL3 among patients with CAD is likely to be multifactorial. Dyslipidemia is the major contributor to CV diseases [12]. The ANGPTL3 deficiency-related hypolipidemic phenotype is driven by enhanced lipoprotein turnover resulting in impaired energy substrate distribution in tissues [9]. Studies in mice and humans have shown that ANGPTL3 acts as a potent inhibitor of LPL, clearing TG-rich lipoproteins from circulation, particularly in the postprandial state [28]. Additionally, ANGPTL3 is an endogenous inhibitor of EL which might regulate HDL-C particles and affect glucose homeostasis [29, 30]. LOF variants in *ANGPTL3* have been associated with decreased plasma TG, LDL-C, and HDL-C levels via loss of LPL and EL inhibition [17, 30]. Furthermore, a study in Ldlr-deficient mice revealed that ANGPTL3 modulates serum LDL-C clearance independently of the LDL receptor [31]. Alternatively, decreased LDL-C levels may be the result of lower LDL precursor and hepatic VLDL particle secretion rates, suggesting that ANGPTL3 may effectively reduce serum LDL-C levels in patients with homozygous familial hypercholesterolemia with a complete LDL receptor-mediated LDL-C uptake deficiency [31]. In the DiscovEHR study, ANGPTL3 LOF mutation carriers had significantly lower circulating TG, LDL-C, and HDL-C (27%, 9%, and 4%, respectively) levels than noncarriers [17]. Moreover, the genetic and therapeutic antagonism of *Angptl3* in mice and *ANGPTL3* in humans has been associated with decreased levels of all major lipid fractions, thereby providing protection from atherosclerotic CV disease [17].

Atherosclerosis of the coronary artery is associated with endothelial dysfunction, adipocyte metabolism dysregulation, and various inflammatory processes [32]. ANGPTL3 has potential atherogenic properties and could directly promote atherosclerosis in humans [15]. ANGPTL3 acts as proangiogenic and could induce angiogenesis *in vivo* via binding of the C-terminal fibrinogen-like domain to the integrin *α*_v_*β*_3_ receptor on vascular endothelial cells, affecting blood vessel formation via the induction of integrin-*α*_v_*β*_3_-dependent endothelial cell migration and adhesion [5]. ANGPTL3 induced angiogenesis with a magnitude comparable to vascular endothelial growth factor-A, which promotes intimal thickening and induces atherosclerosis [33]. Additionally, the association between *ANGPTL3* polymorphisms and coronary plaque is independent of lipids and other confounding variables in MI survivors [34]. Positively associated with plasma ANGPTL3 level and intima-media thickness of the human carotid and femoral arteries is independent of lipids and other classical risk factors, including age, BP, and plasma lipid and glucose levels [15]. All these studies indicate that ANGPTL3 is significantly associated with atherosclerosis and is independent of plasma lipid levels.

The present study has some limitations. First, a limited number of MACE patients, all recruited at a single center, were included. Additionally, lifestyle habits known to influence the occurrence of MACEs, including smoking, alcohol consumption, physical inactivity, and unhealthy diet, were not evaluated and could restrict the study's predictive power. Second, although several medications commonly used by patients with CAD may influence the underlying inflammatory and atherosclerotic status, the present study demonstrated that ACEi, ARB, *β*-blockers, CCB, and fibrates have no impact on circulating ANGPTL3 levels or on new MACE development. However, statin use was significantly associated with a lower occurrence of new MACEs in patients with CAD [35, 36]. Further studies are necessary to clarify the impact of the above medications on serum ANGPTL3 levels and new MACE development in the CAD population. Finally, although we propose an explanation for the mechanism underlying serum ANGPTL3-induced MACEs in patients with CAD, further studies are required before a direct causal relationship can be established between circulating ANGPTL3 levels and development of MACEs in this patient population.

## 5. Conclusion

The present study shows that elevated ANGPTL3 levels represent an independent risk factor for CV events in patients with CAD, with an increased predictive value for MACEs.

## Figures and Tables

**Table 1:** Clinical variables of the 90 coronary artery disease patients according to the serum median of angiopoietin-like protein 3 levels.

Variables	All participants (*n* = 90)	Low ANGPL3 group (*n* = 45)	High ANGPL3 group (*n* = 45)	*P* value
Age (years)	65.51 ± 9.02	67.04 ± 10.04	63.98 ± 7.69	0.107
Height (cm)	161.14 ± 8.18	161.80 ± 7.61	160.49 ± 8.75	0.456
Body weight (kg)	68.61 ± 12.26	69.07 ± 12.46	68.15 ± 12.18	0.724
Body mass index (kg/m^2^)	26.31 ± 3.52	26.29 ± 3.76	26.33 ± 3.30	0.960
Systolic blood pressure (mmHg)	131.08 ± 16.66	129.78 ± 16.65	132.38 ± 16.76	0.462
Diastolic blood pressure (mmHg)	71.99 ± 9.46	73.27 ± 9.62	70.71 ± 9.23	0.202
Total cholesterol (mg/dL)	163.60 ± 32.20	160.09 ± 28.86	167.11 ± 35.21	0.304
Triglycerides (mg/dL)	127.50 (88.75–181.00)	127.00 (91.00–155.50)	128.00 (88.50–201.00)	0.634
HDL-C (mg/dL)	43.81 ± 11.43	43.36 ± 9.25	43.27 ± 13.35	0.758
LDL-C (mg/dL)	95.76 ± 26.61	92.64 ± 25.56	98.87 ± 27.55	0.270
Fasting glucose (mg/dL)	111.00 (95.75–150.50)	111.00 (94.00–167.50)	111.00 (98.50–134.00)	0.865
Blood urea nitrogen (mg/dL)	16.00 (13.00–19.00)	16.00 (14.00–19.00)	16.00 (13.00–19.00)	0.509
Creatinine (mg/dL)	1.10 (0.90–1.30)	1.10 (0.90–1.25)	1.10 (0.90–1.30)	0.687
eGRF (mL/min)	68.53 ± 18.17	68.71 ± 19.77	69.02 ± 18.59	0.936
ANGPTL3 (ng/mL)	222.37 (152.57–320.12)	152.94 (93.10–197.90)	318.40 (278.94–463.04)	<0.001^*∗*^
C-reactive protein (mg/dL)	0.20 (0.14–0.26)	0.17 (0.13–0.20)	0.26 (0.18–0.39)	<0.001^*∗*^
Female (%)	23 (25.6)	9 (20.0)	14 (31.1)	0.227
Diabetes (%)	44 (48.9)	22 (48.9)	22 (48.9)	1.000
Hypertension (%)	70 (77.8)	37 (82.2)	33 (73.2)	0.310
ACE inhibitor use	28 (31.1)	15 (33.3)	13 (28.9)	0.649
ARB use	36 (40.0)	18 (40.0)	18 (40.0)	1.000
*β*-blocker use	54 (60.0)	29 (64.4)	25 (55.6)	0.389
CCB use	30 (33.3)	17 (37.8)	13 (28.9)	0.371
Statin use	67 (74.4)	33 (73.3)	34 (75.6)	0.809
Fibrate use	16 (17.8)	10 (22.2)	6 (13.3)	0.270
One-vessel CAD	37 (41.1)	23 (51.1)	14 (31.1)	0.151
Two-vessel CAD	30 (33.3)	12 (26.7)	18 (40.0)	
Three-vessel CAD	23 (25.6)	10 (22.2)	13 (28.9)	

Values for continuous variables are given as means ± standard deviation and compared by Student's *t*-test; variables not normally distributed are given as medians and interquartile range and compared by Mann–Whitney *U* test; values are presented as number (%), and analysis was performed using the chi-square test. HDL-C, high-density lipoprotein cholesterol; LDL-C, low-density lipoprotein cholesterol; eGFR, estimated glomerular filtration rate; ANGPTL3, angiopoietin-like protein 3; ACE, angiotensin-converting enzyme; ARB, angiotensin-receptor blocker; CCB, calcium-channel blocker; and CAD, coronary artery disease. ^*∗*^*P* < 0.05 was considered statistically significant.

**Table 2:** Clinical variables of the 90 coronary artery disease patients with or without the cardiovascular event.

Variables	Participants without cardiovascular events (*n* = 57)	Participants with cardiovascular events (*n* = 33)	*P* value
Age (years)	65.70 ± 9.29	65.18 ± 8.68	0.794
Height (cm)	161.11 ± 8.70	161.21 ± 7.31	0.953
Body weight (kg)	68.74 ± 12.32	68.38 ± 12.34	0.896
Body mass index (kg/m^2^)	26.39 ± 3.61	26.18 ± 3.41	0.793
Systolic blood pressure (mmHg)	128.82 ± 15.85	134.97 ± 17.55	0.092
Diastolic blood pressure (mmHg)	71.16 ± 9.54	73.42 ± 9.30	0.276
Total cholesterol (mg/dL)	159.65 ± 30.44	170.42 ± 34.45	0.127
Triglycerides (mg/dL)	111.00 (87.50–153.00)	150.00 (90.50–208.00)	0.117
HDL-C (mg/dL)	42.72 ± 9.81	45.70 ± 13.75	0.236
LDL-C (mg/dL)	93.93 ± 26.58	98.91 ± 26.77	0.395
Fasting glucose (mg/dL)	107.00 (96.50–132.50)	111.00 (95.00–181.50)	0.533
Blood urea nitrogen (mg/dL)	16.00 (14.00–19.00)	15.00 (12.00–19.00)	0.176
Creatinine (mg/dL)	1.10 (0.90–1.30)	1.00 (0.90–1.25)	0.187
eGFR (mL/min)	66.36 ± 17.04	72.27 ± 19.69	0.138
ANGPTL3 (ng/mL)	206.67 (110.39–274.38)	318.40 (195.56–490.61)	<0.001^*∗*^
C-reactive protein (mg/dL)	0.16 (0.12–0.21)	0.26 (0.21–0.55)	<0.001^*∗*^
Female (%)	15 (26.3)	8 (24.2)	0.828
Diabetes (%)	25 (43.9)	19 (57.6)	0.210
Hypertension (%)	43 (75.4)	23 (81.8)	0.483
ACE inhibitor use	21 (36.8)	7 (21.2)	0.123
ARB use	21 (36.8)	15 (45.5)	0.422
*β*-blocker use	33 (57.9)	21 (63.6)	0.592
CCB use	17 (29.8)	13 (39.4)	0.353
Statin use	47 (82.5)	20 (60.6)	0.022^*∗*^
Fibrate use	8 (14.0)	8 (24.2)	0.222
One-vessel CAD	33 (57.9)	4 (12.1)	<0.001^*∗*^
Two-vessel CAD	14 (24.6)	16 (48.5)	
Three-vessel CAD	10 (17.5)	13 (39.4)	

Values for continuous variables are given as means ± standard deviation and compared by Student's *t*-test; variables not normally distributed are given as medians and interquartile range and compared by Mann–Whitney *U* test; values are presented as number (%), and analysis was performed using the chi-square test. HDL-C, high-density lipoprotein cholesterol; LDL-C, low-density lipoprotein cholesterol; eGFR, estimated glomerular filtration rate; ANGPTL3, angiopoietin-like protein 3; ACE, angiotensin-converting enzyme; ARB, angiotensin-receptor blocker; CCB, calcium-channel blocker; and CAD, coronary artery disease. ^*∗*^*P* < 0.05 was considered statistically significant.

**Table 3:** Hazard ratio for cardiovascular events by multivariable Cox regression of angiopoietin-like protein 3 levels among the 90 patients with coronary artery disease.

ANGPTL3 (ng/mL)	Unadjusted		Model 1		Model 2		Model 3	
	HR (95% CI)	*P* value	HR (95% CI)	*P* value	HR (95% CI)	*P* value	HR (95% CI)	*P* value
Per 1 ng/mL ANGPTL3 increase	1.003 (1.002–1.004)	<0.001^*∗*^	1.003 (1.002–1.005)	<0.001^*∗*^	1.004 (1.003–1.006)	<0.001^*∗*^	1.003 (1.000–1.005)	0.026^*∗*^

Model 1 is adjusted for age, gender, and body mass index. Model 2 is adjusted for Model 1 variables and for diabetes mellitus, hypertension, fasting glucose, total cholesterol, triglyceride, low-density lipoprotein cholesterol, and estimated glomerular filtration rate. Model 3 is adjusted for Model 2 variables and for C-reactive protein, statin used, and severity of coronary artery disease. ^*∗*^*P* < 0.05 was considered statistically significant after Cox regression analysis. ANGPTL3, angiopoietin-like protein 3; HR, hazard ratio; and CI, confidence interval.

## Data Availability

The data underlying this study are available from the corresponding author on reasonable request.
